# Will the Policy Instruments Mix Promote the Facility Input of Care Institutions for Older People in China?

**DOI:** 10.3389/fpubh.2022.840672

**Published:** 2022-06-01

**Authors:** Fen Zhang, Xiaodong Di, Xiao Yang, Xiaotian Yang, Quanbao Jiang, Changhong Yuan

**Affiliations:** ^1^School of Public Policy and Administration, Xi'an Jiaotong University, Xi'an, China; ^2^School of Management, Xi'an Jiaotong University, Xi'an, China; ^3^School of Economics and Management, Dalian University of Technology, Dalian, China

**Keywords:** care institutions for older people, facility input, policy support, policy instrument mix, comprehensiveness, consistency, balance

## Abstract

Promoting the facility input of care institutions for older people is the key to the development of the care for older people. With a steady increase in the proportion of older people in China, institutional care services are considered as an important tool for older people. Policies such as government bed subsidies and tax incentives are accelerating the development of institutional care services. However, when the care for older people related policy instrument mixes lacks overall comprehensiveness, consistency and balance, the policy instrument mixes may become a “policy mess”, in turn, affecting the development of care institutions for older people. Studies focusing on the combined effects of different characteristics of a policy instrument mix are scarce. To understand how to better use the policy mix to facilitate the care for older people, it is necessary to analyze the characteristics of care policies for older people and its role in the construction of care institutions for older people. Therefore, this study analyzes the impacts of comprehensiveness, consistency, and balance of policy instruments on the facility input of care institutions for older people. An empirical analysis of related policies from 2011 to 2019 in China shows that a synergetic effect exists between the policy instrument mix and the facility input of care institutions for older people. This study points that the comprehensiveness and consistency of the policy instrument mix positively affect the facility input of care institutions for older people, while the impact of balance is not significant. It not only provides feasible policy suggestions for China's policy-making departments to optimize the care for older people related policies, but also helps care institutions for older people further understand the characteristics of policy portfolios and realize sustainable development.

## Introduction

As of November 2020, China recorded 264.02 million people aged over 60, accounting for 18.7% of the total population, and 190.64 million people aged over 65, representing 13.5% of the overall population. Care for older people has become a significant issue affecting social development in China (1, 2). With a decline in the birth rate and family care functions, community care and home care have failed to provide older people high-quality care services. Therefore, institutional care has become an irreplaceable “support” status in care for older people models with the rapid increase in demand for care for older people in the coming decades (3, 4). Institutional care for older people refers to public and private care institutions that provide places for care services for older people, including nursing homes and elderly apartments. The staff at care institutions for older people provides all-day accommodation and care services for older people. The government, charity institutions, or families bear the expenses of care services for older people.

Promoting the facility input of care institutions for older people is of great significance to meet the needs and services of older people, better promote the development of care service, and ultimately solve the problem about care for older people. According to the evaluation index system of the “Thirteenth Five-Year Plan for the Development of National Aging and the Construction of the Older People Care System” the facility input of care institutions for older people is mainly measured by indicators such as the number of older people care beds and nursing beds operated by the government. Therefore, in our study, we use the number of older people's care beds to reflect the facility input of care institutions. By 2019, the number of institutional care beds for older people has reached 4.385 million in China, but it is still unable to meet the needs of older people. However, the construction of care institutions for older people has the characteristics of high capital investment and a long payback period (5), which directly results in a severe shortage of social care for older people facility input (6). Consequently, the public has low confidence in investing in the care industry for older people, and firm's responses to provide facility of care institutions for older people may not get momentum, if there is no support of policymakers (7).

Given that relying solely on the market is far from satisfying the demand of care for older people in China, the government has promulgated many crucial policies to help promote the development of care institutions for older people. Policymakers can contribute to stimulating firms' willingness to invest through issuing related policies. Therefore, multiple policy instruments can be combined, which can be called a “policy instrument mix” (8, 9). Policy instrument mix is the combination of different policy instruments that belong to the same policy area (7). Chinese government has promulgated many policies to promote the development of care industry for older people. For example, in December 2016, with the growth of the care service market for older people, an increasing number of policy instruments have been created and applied, and the effectiveness of policy instruments has become increasingly significant. The institutional care service system has become comprehensive, and service quality has improved significantly. In 2019, the “Opinions of the General Office of the State Council on Promoting the Development of Elderly Care Services” further indicated that the administrative approval process should be simplified; social organizations should be encouraged to participate in the construction and operation of care institutions for older people; the development of care institutions for older people should be accelerated.

Among these policies, many policy instruments, such as fiscal and taxation, construction standards, approval standards, and fire safety, are combined to help promote the development of care institutions for older people. The mix of policy instruments promulgated by the government is critical to the development of care institutions for older people. However, with the steady growth in care service policies for older people and the increase in policy promulgators and joint departments, policies supporting for the care of older people have gradually shown the characteristics of policy crowdedness, poor policy convergence, and chaotic standards (10). Indeed, when the mix of instruments lack overall comprehensiveness, consistency and balance, the policy instrument mixes may become a “policy mess” (7, 9, 11).

The complex policy mix leads to issues such as unbalanced facility input as bed numbers provided by care institutions for older people and a mismatch between supply and demand, hindering the development of the care service industry for older people. Recently, scholars have recognized the importance of a policy instrument mix on firms' strategies (7) and called for more research to investigate the influence of policy instrument mix (9, 12). However, the evidence of such research which aims at studying the effectiveness of different characteristics among policy instrument mix is very limited (13). Therefore, it is imperative to analyze the impact of the characteristics of elderly care policy instruments on elderly care, to develop policies supporting for the development of institutional care for older people. However, such kind of literature on this topic is scarce (14). To the best of our knowledge, very few studies have investigated the effects of comprehensiveness, consistency, and balance of the policy instrument mix on the facility input of care institutions for older people.

This study seeks to investigate the existing issues within the policy instrument mix, provide suggestions for the government to revamp relevant policies, and guide care institutions for older people to make facility input decisions, to improve the utilization and accessibility of institutional care facilities. From the perspective of institutional elderly care policy instruments, based on elderly care service policies issued by the State Council and various ministries and commissions of the State Council between 2011 and 2019, our study investigated the impact of the characteristics of the policy instrument mix on the facility input of care institutions for older people. First, from the perspective of policy instrument mix, this study categorizes elderly care service policy instruments into three types: supply-based, demand-based, and environmental-based. Furthermore, it calculates the comprehensiveness, consistency, and balance of the policy instrument mix to characterize the interrelationships between policies. Subsequently, it builds a model to analyze the impact of the characteristics of the policy instrument mix on the facility input of care institutions for older people. Finally, according to the empirical results, it recommends feasible suggestions for optimizing the relevant policy design of institutional care from different policy instrument mix perspectives, the application of policy instruments, and other aspects. The policy instrument mix plays an important role in the development of care institutions for older people by analyzing how the characteristics of elderly care policy instruments affect institutional care facilities. It not only provides feasible policy suggestions for China's policy-making departments to optimize the resource investment of care institutions for older people, but also helps these institutions further understand the characteristics of policy portfolios and realize sustainable development.

## Literature and Theory

### Policy Instrument Mix of Care Institutions for Older People

The phrase “policy mix” emerged and gained popularity in the economic policy literature from the 1960s to the early 1990s. It has been extended to other areas of public policy to explore the interaction between different policy instruments to achieve a specific goal or outcome (15). In a shift from a single policy, there are interactions among multiple policies included in the policy mix (14), which will produce deeper and differentiated policy outcomes. Some scholars have pointed out that policy mix refers to a combination of different policy instruments, and the interactive relationship between policy instruments is the basis of the policy combination (16). Accordingly, studies focusing solely on the interaction of instruments should, specifically, refer to the term “instrument mix” (17). Therefore, a policy instrument mix is a combination of policy indicators that interact with each other (18). Similarly, an care for older people policy instrument mix is a composite set of policy instruments that interact with each other, mainly including construction standards, service requirements, and resource input.

As for the classification of policy instruments, Bemelmans suggested that policy instruments can be divided into regulatory, economic, financial, and soft instruments (19). Howlett and Ramesh categorized policy instruments into four types: mandatory, market, information transmission, and voluntary (20). Chen believed that policy instruments included market instruments, business technologies, and social measures (21). However, in the field of elderly care services, most scholars categorize policy instruments into environmental, supply-based, and demand-based policies (22). Yue divided the integrated care policy for older people into supply-side, demand-side, and environmental policy instruments, and found that environmental policy instruments are the most frequently used, supply-side policies are preferred, while demand-side policy instruments are relatively inadequate (23). Xiu found that China's local care policies for older people can be classified into supply, demand, and environmental policy instruments (24). Mature regions use more environmental policy instruments, which can help stimulate care institutions for older people to provide better care for older people.

Policy instruments are practical means and methods adopted by decision-makers to achieve policy goals. This study adopted the policy instrument model of Rothwell and Zegveld (22). This model weakens the mandatory characteristics of policy instruments, and has a clear market orientation, in line with the current development direction of institutional elderly care services. Based on this model, we constructed the analysis framework as “policy instruments and facility input of care institutions for older people.” Care-related policy instruments for older people are divided into demand-based, supply-based, and environmental policy instruments. Demand-based policy instruments reflect the influence of policies on the development of institutional care services and reduce market barriers through government procurement, service outsourcing, market shaping, and international exchanges. Supply-based policy instruments are manifested as the driving force of policies for the development of care for older people and help the supply-side reform of elderly care services through capital, technology, and facility investments. Environmental policy instruments reflect the guiding role of policies for the development of institutional care services, mainly through tax incentives, administrative supervision, and other economic policies, to create an appropriate environment for guiding the development of care for older people. In addition, through the text analysis of the policies we collected, interactive comparisons and the discussion among our group members, we classified the content and key words corresponding to each policy instruments category. The specific institutional care policy classification is shown in Table 1.

**Table 1:** Classification and content description of policy instruments.

**Types of policy instruments**	**Content**	**Key words**
Demand-based policy instruments	Government Procurement	Government purchasing services
	Service outsource	Social capital
	Market shaping	Market cultivation
	International exchange	Global cooperation
Supply-based policy instruments	Talent development	Education training
	Capital investment	Funding
	Technology investment	Technology R & D
	Facility investment	Supporting facilities
	Information service	Information platform
Environmental policy instruments	Tax incentives	Tax deduction
	Technical Support	Industry-University-Research Cooperation
	Land Policy	Land security
	Administrative measures	Simplify the approval process
	Other economic policies	Water and electricity fee reduction

### Impact of Policy Instrument Mix on Care Services for Older People

As part of its policy tasks and strategies, the government has attempted to establish a care framework for older people, aiming to promote social forces as market players, open up the care service market for older people, improve the consumption capacity of both households and individuals, and improve service quality. From the perspective of policy instrument mix, investigating the effect of the characteristics of the policies issued by the government on the development, management, and supervision of the elderly care industry is helpful to analyze each developing stage of care service for older people and to understand the key points of care institutions for older people in each growth period.

Policy instrument mixes offer a new perspective for understanding local care policies for older people. They are used by policymakers to achieve policy goals. The policy instruments that can support elderly care policy refer to the ways to realizing an effective and fair supply of care services for older people (24). In the field of care services for older people, most scholars concerned about the impact of a single policy such as tax policy and subsidy policy on elderly care services. Kim found that the subsidy policy promoted equity of access to public long-term care services, and the subsidized users were more likely to choose institutionalized care (25). Song theoretically verified the importance of subsidy policies in stimulating the private supply of care for older people (7). Kpessa found that three public policies—pension policy for retirement income security, exemptions granted to older people under the national health insurance scheme, and the cash transfer program meant for poverty alleviation—could improve the quality of care services for older people (26). Zhang indicated that long-term care policy, as an outcome of policy transfer, served as a rational tool to determine China's aging problems (27).

The selection of elderly care policy instruments should correspond to the elderly care service system and adapt to the overall goal of the elderly care service system. Policy instruments are used by governmental organizations as tools to influence firms' strategic choices (8). In this study, care service policy instruments for older people are categorized into environmental, supply-based, and demand-based policy instruments. Considering the existing interaction effects between policy instruments, the policy instrument mix has its own characteristics, such as comprehensiveness, consistency, and balance (22).

The comprehensiveness of the care policy instrument mix for older people refers to the range of the application of elderly care-related policies (19). Comprehensiveness measures the diversity of policy tools and policy objectives. The stronger the comprehensiveness is, the more types of care policy instruments for older people involved in stimulating the facility input of care institutions for older people. The comprehensive use of policy instruments can promote the effectiveness of the elderly care system and address potential problems in the system. In addition, the adoption of a wide range of care policies for older people can provide an appropriate environment for the development of the industry and more effectively activate the enthusiasm of care institutions for older people to introduce additional facilities (12).

The consistency of the care for older people policy instrument mix measures the synergy between policies within the policy portfolio and reflects the differences between policies (17). The policy portfolio with high consistency has less conflict among policies and even has a certain synergy. The consistency and coordination between policies can provide a favorable institutional environment for care institutions for older people and effectively promote the development of care services for older people. Individual policy "fighting alone” has a limited impact on promoting the development of the care for older people industry (11). Therefore, the government often promulgates a variety of policy tools or policy objectives to realize the combination linkage between different policies through coordinated allocation, to make the policy combinations more effective. The coordination and consistency between policies in the policy portfolio can increase the effectiveness of policies to a greater extent and promote the effectiveness and accessibility of care-related policies for older people (15). Increasing the consistency of the policy instrument mix will strengthen elderly care institutions' confidence in committing more resources.

The balance of care policy instrument mix for older people measures the balance of the content focus and application frequency of policy tools under different policy mix (17). For example, the content focus of older people care policy instruments include tax policy, financial policy, land policy and other policy, equilibriums among these content focus reflect the balance of care policy instrument mix. A balanced policy mix may help form a more reliable and stable policy framework (9), and subsequently promote the facility input of care institutions for older people, while an unbalanced policy instrument mix may reduce the expectation of care institutions for older people for the market and reduce the facility input. The imbalanced use of policy instruments such as the economic policy, platform construction and social organization cultivation, will greatly limit the formation of the care for older people service market and inhibit the supply of care for older people service resources. If the balance of care for older people policy instrument mix can be enhanced, the facility input of care institutions for older people are promoted. In conclusion, the following assumptions were made.

Based on the above analysis, this study advances the following hypotheses.

Hypothesis 1: The comprehensiveness, consistency, and balance of the policy instrument mix promote the facility input of care institutions for older people.
Hypothesis 2: The comprehensiveness, consistency, and balance of demand-based policy instrument mix promote the facility input of care institutions for older people.
Hypothesis 3: The comprehensiveness, consistency, and balance of supply-based policy instrument mix promote the facility input of care institutions for older people.
Hypothesis 4: The comprehensiveness, consistency, and balance of environmental policy instrument mix promote the facility input of care institutions for older people.

## Policy Instrument Mix

### Policy Quantification and Calculation

This study investigates the comprehensiveness, consistency, and balance of China's care policy instruments for older people from the perspective of the interaction between policy instruments and the interrelationship between policies.

First, we analyzed the effectiveness of the policy instrument mix. Thereafter, we accumulated the scores of a certain indicator among the various policy instruments of the newly promulgated policies each year, and obtained the total score (TS) of all indicators of a certain care for older people policy.


(1) $$\begin{array}{r} TS_{t}=\overset{N}{\underset{j=1}{\sum }}PG_{tj}\times P_{tj} \end{array}$$

Where *j* represents the policy; *N* is the total number of policies in year *t*; *PG* represents the score of a certain index; the value of *PG* changes according to different evaluation criteria and different research contents. We here followed the existing literature and defined *PG* as 1, which means that all indicators share the same level importance (28). *P* reflects the effectiveness of the policies, ranging from 1 to 5. The specific measurement standards are that five points represent promulgated laws by the National People's Congress and its standing committee. Four points represent regulations and orders of various ministries and commissions promulgated by the State Council. Three points illustrate interim regulations, opinions, methods, and decisions promulgated by the State Council; regulations of various ministries and commissions; and regulations promulgated by provincial administrative units. Two points denote temporary regulations, methods, opinions, and plans of various ministries and commissions; opinions and regulations promulgated by various provincial administrative units. One point typifies notices and announcements.

We here took a very commonly used measure to calculate policy quantification (12, 22, 23). Based on the calculation of *TS*, the comprehensiveness, consistency, and balance of different policy instrument mixes within the same dimension and different policy indicator mixes within the same policy were measured.

Comprehensiveness refers to the range of the policy applications. Multiple policy measures must be applied simultaneously in the development of care services for older people. It was obtained by calculating the cumulative sum of the indicator scores of the different policy instruments.


(2) $$\begin{array}{r} COM=\overset{l}{\underset{r=1}{\sum }}TS_{t}^{r} \end{array}$$

Where, *COM* represents comprehensiveness; *r* represents the indicator within the policy instrument; *l* is the total number of indicators within the policy instruments.

Consistency implies that there are fewer conflicts between different policy instrument mixes. This study used the method of calculating the cosine of the vector angle to reflect the consistency of the policy instrument mix.


(3) $$\begin{array}{r} CON=\left(\overset{l}{\underset{r=1}{\sum }}TS\right)\times \frac{\overset{k}{\underset{i=1}{\sum }}\overset{k}{\underset{j=1}{\sum }}\cos\left(X_{t}^{i},X_{t}^{j}\right)}{N\times \left(N-1\right)/2} \end{array}$$

(4) $$\begin{array}{r} \cos\left(X_{t}^{i},X_{t}^{j}\right)=\frac{\overset{l}{\underset{r=1}{\sum }}\left(x_{t}^{ir}\times x_{t}^{jr}\right)}{\sqrt{\overset{l}{\underset{r=1}{\sum }}{\left(x_{t}^{ir}\right)}^{2}}*\sqrt{\overset{l}{\underset{r=1}{\sum }}{\left(x_{t}^{jr}\right)}^{2}}},\forall i\neq j \end{array}$$

Where, *CON* represents consistency; *i* and *j* are any two policies promulgated in year *t*; the instrument indicator vector of a certain policy is $X_{t}^{i}=\left(x_{t}^{i1},x_{t}^{i2},\cdot \cdot \cdot ,x_{t}^{il}\right)$; $\cos\left(X_{t}^{i},X_{t}^{j}\right)$ is the vector cosine value of the two policies. The larger the cosine value, the stronger the consistency.

The balance of care for older people policy instrument mix measures the balance of the policy content focus and application frequency of policy tools under different policy mix. Unbalanced indicators hinder the development of care services for older people. Before measuring balance, we first calculated the correlation index between indicators in different policy instruments.


(5) $$\begin{array}{r} RE_{t}^{d}={\left[\frac{\left|TS_{mt}-TS_{nt}\right|}{\sqrt{TS_{mt}+TS_{nt}}}\right]}^{-1},\forall m\neq n \end{array}$$

Where *RE* is the correlation coefficient between different policy instrument indicators, *m* and *n* represent different policy instrument indicators; *d* is a combination of two policy instrument indicators.


(6) $$\begin{array}{r} BAL_{t}=\sqrt{\frac{{\overset{n^{*}}{\underset{d=1}{\sum }}\left(RE_{t}^{d}-\frac{\overset{n^{*}}{\underset{d=1}{\sum }}RE_{t}^{d}}{n^{*}}\right)}^{-1}}{n^{*}}} \end{array}$$

Where, *BAL* represents balance; represents the total number of pairs of policy indicators.

### Data Sources and Calculation Results

Considering the period of high-profile publications and the binding force of policy texts comprehensively, we selected normative policy texts from 2011 to 2019 for the analysis, and used the Peking University Magic Law Database and the official websites of the State Council and various ministries and commissions to search “care for older people,” “care services for older people,” “care for older people system,” “home care,” “community care,” “combined medical care and care for older people,” “smart care,” “Internet care,” “aging,” and other keywords. Overall, 216 national policies related to institutional care for older people were identified. Finally, by analyzing the policy content, 172 institutional care policies were selected for analysis based on the time, effectiveness, revocation, and overlap of the policy.

**Figure 1:**
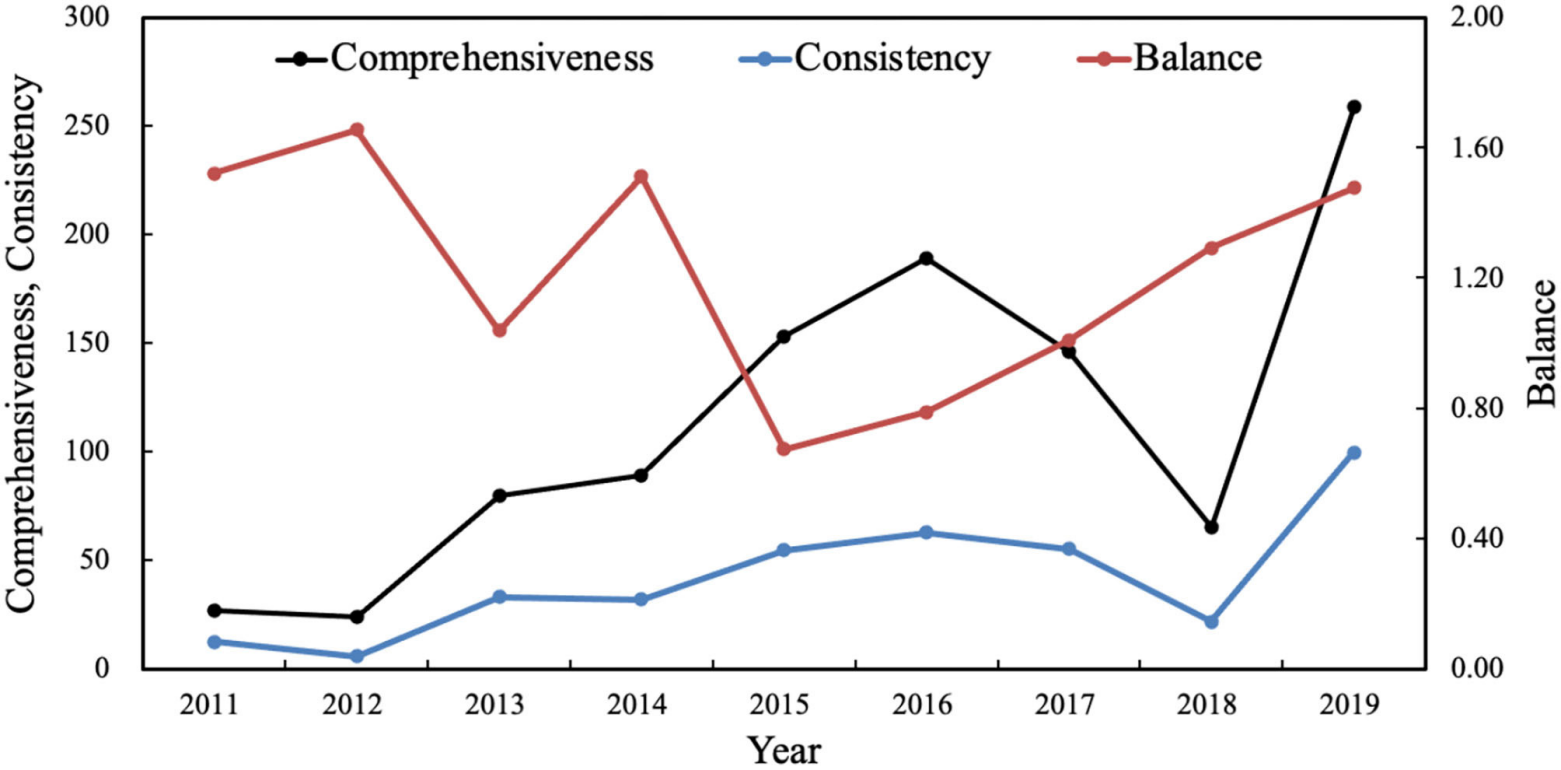
Characteristic trends of policy instrument mix of institutional care for older people in 2011–2019.

According to the above formula, the characteristic trend of the institutional care policy instrument mix is finally obtained, as shown in Figure 1.

In Figure 1, the comprehensiveness and consistency of the policy instrument mix are very close. It shows an increasing trend by year from 2011 to 2016. This demonstrates that China has increased the promulgation of care policies for older people in recent years and attaches importance to care services for older people. In 2017 and 2018, the comprehensiveness and consistency of the care policy instruments for older people declined marginally. This can be attributed to the promulgation of the “Notice of the State Council on Printing and Distributing the National Aging Career Development and Pension System Construction Plan for the 13th Five-Year Plan,” which emphasizes the comprehensive coordination of care for older people service. The development of care services for older people has shifted from comprehensive construction to improving quality and efficiency.

Meanwhile, the balance of policy instruments has increased annually from 2015, indicating that demand-based, supply-based, and environmental policies are coordinated with each other, and that the exploration in institutional care services has achieved positive results.

**Figure 2:**
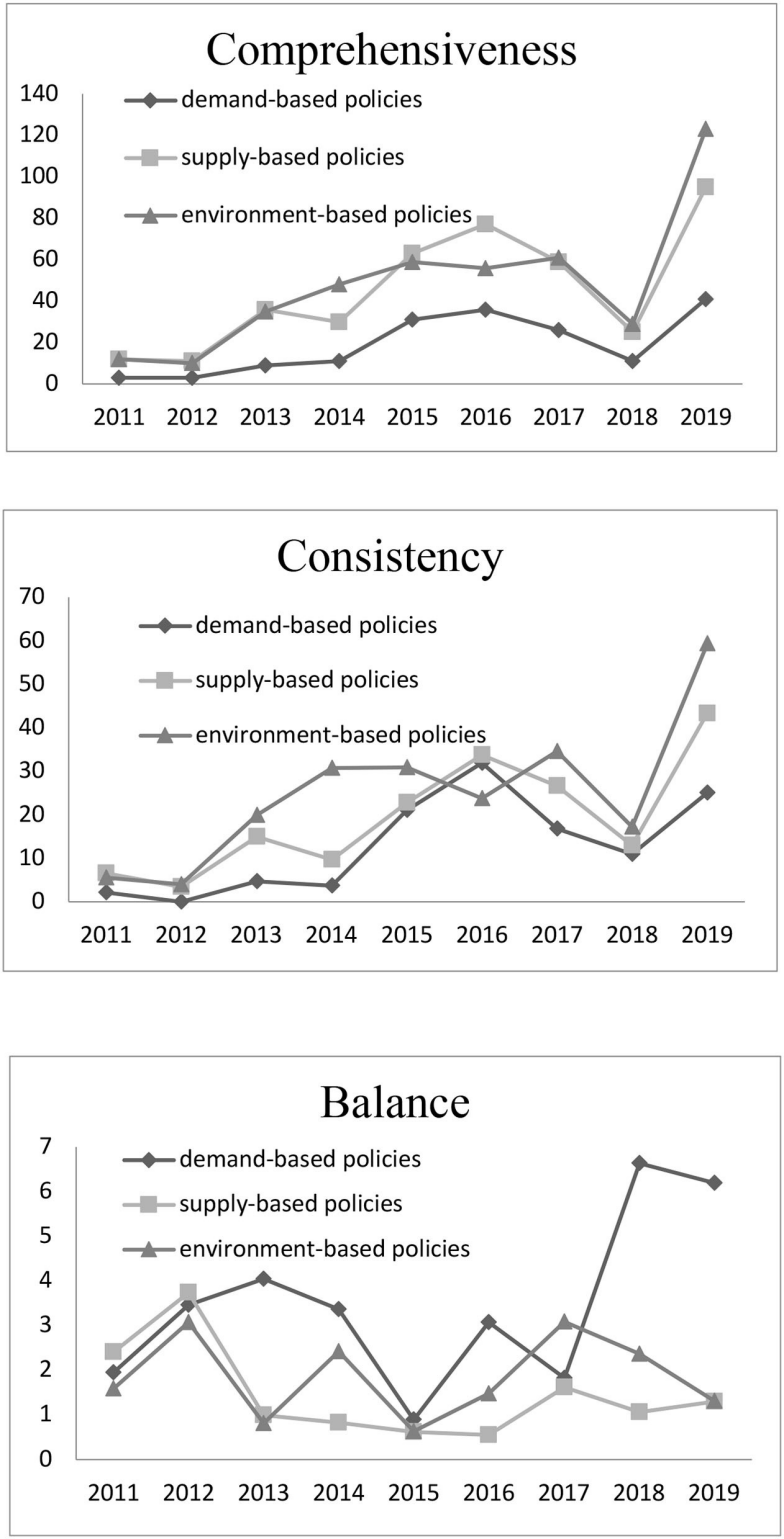
Changes in the characteristics of different policies within the same policy instrument mix from 2011–2019.

In Figure 2, the comprehensiveness and consistency trends are relatively similar, and the demand-based policy index is the smallest. It indicates that China's institutional care services mainly rely on supply-based and preferential policies to drive the development of care services for older people, ignoring the market demand for care services for older people.

The balance of the three policy instrument mixes presents a fluctuating development state. Overall, the balance of the demand-based policy is higher, and that of supply-based policy is the lowest. Although demand-based policy is not the main policy instrument for care services for older people in China, its development is balanced. It indicates that the government and the market have better coordination in the development of care services for older people. The balance of demand-based policy unexpectedly increased in 2018 and 2019, which was mainly related to policies such as the development of smart care and the opening of the care for older people service market.

## Impact Results

To explore the impact of the policy instrument mix on the facility input of care institutions for older people, this study collected provincial panel data for 2011–2019, using the 2012–2020 China Statistical Yearbook, Local Statistical Yearbook, China Civil Affairs Statistical Yearbook, the 2011–2019 provincial and municipal statistical bulletins, China's economic and social development statistical database, and EPS database.

### Variables

We considered the facility input of care institutions for older people as the dependent variable. Affected by policies, the state's subsidies to care institutions for older people are mostly bed subsidies; the core asset of care institutions for older people is dependent on the number of beds. Therefore, this study used bed numbers (EB) to measure the facility input of care institutions for older people, following the China Statistical Yearbook, the unit of EB's measurement is ten thousand.

The independent variables included the comprehensiveness, consistency, and balance of the policy instrument mix (PM).

Based on research on the factors affecting the facility input of care institutions for older people, we considered the following control variables: (1) GDP per capita (PG), which measures the social and economic development status. The unit of PG's measurement is yuan. The better the economic development, the more adequate the facility input of care institutions for older people. To further eliminate the influence and error of different dimensions between variables, this study employed the logarithmic form of GDP per capita (lnPG). (2) Fixed asset investment (FI): The investment reflects the intensity of investments in social resource construction in various regions. If the investment is higher, more resources are used for the construction of institutions' care services. This study employed the logarithmic form of investment (lnFI). (3) Old-age dependency ratio (OR): If the old-age dependency ratio is high, the demand for facilities for care institutions for older people is higher, so the proportion of social capital invested in care institutions for older people is greater. (4) Pension per capita (AE): This was measured as the ratio of urban employee pension insurance expenditure to the number of urban employee retirees at the end of the year. The higher the average pension per capita, the greater the income and demand that older people can use for institutional care. The unit of AE's measurement is ten thousand yuan. (5) Education level (EL): This was measured by the proportion of ordinary college graduates to the total population. The higher the level of social education, the greater is the willingness to receive social care services for older people. The larger the facility input of care institutions for older people, the more the amount of resource required.

The descriptive statistical analysis of the above variables is shown in Table 2.

**Table 2:** Descriptive statistical analysis of related variables.

**Variables**	**EB**	**P G**	**OR**	**AE**	**EL**	**FI**
Number	279	279	279	279	279	279
Average	12.88	54,021.61	13.82	3.11	0.25	17,621.89
Median	10.40	46,674	13.64	2.82	0.24	14,222.22
Max	48.90	164,220	23.82	10.73	0.56	57,466.03
Min	0.10	16,413	6.71	1.43	0.10	516.31
Standard deviation	10.61	26,225.68	3.54	1.22	0.08	12,866.94

Based on the calculation results, the comprehensiveness and consistency of the policy instrument mix showed an increase from 2011 to 2016, a decline from 2017 to 2018, and a steady growth from 2019. As for the bed numbers provided by care institutions for older people in China, it increased from 3.534 million in 2011 to 3.788 million in 2016, from 3.836 million in 2017 to 3.795 million in 2018, then increased to 4.385 million in 2019. From the above descriptions, the changing trend between policy mix characteristics and the bed numbers provided by care institutions for older people seems consistent. Therefore, according to the correlation between the facility input of care institutions for older people and the policy instrument mix, in addition, building on the reviewed literature on the related area (9, 12), we employed the following regression model to evaluate the impact of policy mix characteristics on organizations' decisions:


$$\begin{array}{rcl} EB_{it} & = & \alpha_{0}+\alpha_{1}EB_{it-1}+\alpha_{2}PM_{it}+\alpha_{3}\ln\;FI_{it}+\alpha_{4}\ln\;PG_{it} \end{array}$$

(7) $$\begin{array}{rc} + & \alpha_{5}AE_{it}+\alpha_{6}OR_{it}+\alpha_{7}EL_{it}+\varepsilon_{it} \end{array}$$

Where α_*j*_ (*j* = 1, 2, 3…) is the parameter coefficient; *i* represents the area; *t* represents the time, and εitis the model error.

### Results

In the case of excluding multicollinearity, considering that there are lags and missing variables of the explained variables in the model, the issues of weak instrumental variables, and potential endogeneity which may result from omitted variables, such as economic development level, financial revenue and expenditure level, this study adopted the system generalized method of moment estimation (SYS-GMM) to calculate the parameter of formula (1). Here, we treated the lag of the facility input of care institutions for older people as instrumental variable. SYS-GMM increases the effectiveness of instrumental variables in the form of zero-filling through complex matrix transformations, and simultaneously estimates the level and difference equations of explanatory variables. It not only overcomes the phenomenon of weak instrumentalization of traditional instrumental variables, but also improves the validity and consistency of the estimated parameters.

#### The Policy Instrument Mix of Different Types

We considered the comprehensiveness, consistency, and balance of policy instruments as independent variables. The results are shown in Table 3.

**Table 3:** The impact of instrument mix of different policies on the facility input of care institutions for older people.

**Independent variables**	**Comprehensiveness**	**Consistency**	**Balance**
PM	0.01*** (24.39)	0.02*** (31.20)	−0.06 (−0.41)
L.EB	0.19*** (30.93)	0.17*** (12.31)	0.19*** (17.34)
lnFI	−2.25*** (−3.46)	−2.59*** (−4.76)	−2.83*** (−5.10)
lnPG	1.03 (1.13)	1.51 (1.91)	1.56 (1.74)
AE	0.43*** (6.19)	0.45*** (13.42)	0.53*** (6.95)
OR	0.06 (1.36)	0.01 (0.13)	0.15** (2.75)
EL	−58.45*** (−11.42)	−57.13*** (−13.83)	−52.92*** (−13.34)
C	31.30*** (4.69)	30.30*** (4.68)	30.70*** (5.60)
Wald	2105.67 (0.00)	4453.32 (0.00)	3978.94 (0.00)
Sargan (P)	24.06 (0.68)	24.28 (0.67)	28.92 (0.42)
AR1 (P)	−2.29 (0.02)	−2.25 (0.02)	−2.34 (0.02)
AR2 (P)	−1.00 (0.32)	−1.13 (0.26)	−1.10 (0.27)

*T-values are in parentheses.*

*** *Means significant at the 0.1% level, and*

** *means significant at the 1% level*.

The results of the joint test of the parameters show that the parameters of Models 1–5 in Table 2 are distinctly significant. It indicates that the model setting is reasonable and effective. The results of the Arellano-Bond test show the residuals of all models show a first-order autocorrelation, no second-order autocorrelation at a significance level of 5%, and the non-significant Sargan statistics. It illustrates that the instrumental variables used in each model are all valid.

From the perspective of independent variables, the comprehensiveness and consistency of policy instrument mix of different types have a positive impact on the facility input of care institutions for older people. It indicates that the synergy between policies is enhanced with the number and range of care policies for older people in China, which is conducive to promoting the increase of care institutions. The balance of different types of policy instruments has a negative effect on the facility input of care institutions for older people. This shows that the care for older people service system becomes complete when the indicators of the supply-based, demand-based, and environmental policies are balanced, which can inhibit the blind facility input of care institutions for older people driven by policies.

#### The Policy Instrument Mix of the Same Type

Taking the comprehensiveness, consistency, and balance of the three policy instruments of demand-based, supply-based, and environment-based policies as independent variables, the results are shown in Table 4.

**Table 4:** The impact of same-type policy instrument mix on the facility input of care institutions for older people.

	**Demand-based policies**			**Supply-based policies**			**Environment-based policies**		
	**Comprehensiveness**	**Consistency**	**Balance**	**Comprehensiveness**	**Consistency**	**Balance**	**Comprehensiveness**	**Consistency**	**Balance**
PM	0.01*** (8.74)	0.00 (0.23)	0.32*** (19.82)	0.02*** (34.91)	0.05*** (30.07)	−0.08* (−2.12)	0.01*** (24.24)	0.03*** (19.39)	−0.55*** (−27.66)
L.EB	0.17*** (12.10)	0.18*** (22.32)	0.15*** (15.11)	0.20*** (29.38)	0.21*** (28.99)	0.17*** (10.38)	0.15*** (13.36)	0.13*** (10.06)	0.15*** (16.04)
lnFI	−2.60*** (−4.37)	−2.84*** (−5.59)	−1.75*** (−4.08)	−2.45*** (−3.67)	−2.41*** (−3.99)	−2.72*** (−4.53)	−1.98** (−2.70)	−2.91*** (−5.42)	−3.77*** (−7.01)
lnPG	1.19 (1.11)	1.69* (2.27)	−0.29 (−0.33)	1.07 (1.14)	1.49 (1.81)	1.42 (1.49)	0.40 (0.35)	1.21 (0.88)	3.08** (3.21)
AE	0.50*** (7.36)	0.47*** (6.14)	0.30** (3.27)	0.47*** (7.11)	0.37*** (4.20)	0.52*** (7.41)	0.47*** (15.87)	0.50*** (8.90)	0.63*** (20.23)
OR	0.09 (1.95)	0.13* (2.47)	0.04 (1.86)	0.07 (1.36)	0.00 (0.01)	0.11* (2.10)	0.02 (0.52)	0.04 (0.93)	0.13** (2.97)
EL	−48.82*** (−7.92)	−51.75*** (−10.59)	−40.01*** (−9.01)	−57.82*** (−12.95)	−60.03*** (−14.57)	−52.17*** (−12.76)	−54.13*** (−14.25)	−51.12*** (−14.27)	−54.69*** (−16.08)
C	31.36*** (5.12)	28.67*** (5.37)	38.74*** (6.12)	32.60*** (5.17)	28.74*** (3.94)	31.16*** (5.22)	35.84*** (4.94)	35.98*** (3.58)	22.95*** (3.36)
Wald	1,117.81 (0.00)	3,135.76 (0.00)	14,275.32 (0.00)	2,855.40 (0.00)	3,397.82 (0.00)	2,287.02 (0.00)	8,644.88 (0.00)	5,426.48 (0.00)	5,491.97 (0.00)
Sargan (P)	27.43 (0.50)	28.51 (0.44)	28.36 (0.45)	25.15 (0.62)	23.92 (0.69)	27.67 (0.48)	23.07 (0.73)	25.99 (0.57)	21.65 (0.80)
AR1 (P)	−2.24 (0.02)	−2.31 (0.02)	−2.39 (0.02)	−2.27 (0.02)	−2.26 (0.02)	−2.31 (0.02)	−2.25 (0.02)	−2.21 (0.03)	−2.2 (0.03)
AR2 (P)	−1.13 (0.26)	−1.1 (0.27)	0.53 (0.60)	−0.95 (0.34)	−0.68 (0.50)	−1.16 (0.25)	−1.35 (0.18)	−1.54 (0.12)	−1.72 (0.09)

*T-values are in parentheses.*

*** *Means significant at the 0.1% level,*

** *means significant at the 1% level, and*

* *means significant at the 5% level*.

In Table 4, the comprehensiveness of demand-based, supply-based, and environmental policies has a positive impact on the facility input of care institutions for older people. It indicates that the higher effectiveness of policy institutions is beneficial to the facility input of care institutions for older people. The consistency of the supply-based and environmental policies has a positive impact on the facility input of care institutions for older people. It suggests that a consistent policy is conducive to the construction and operation of care institutions for older people. The impact of the consistency of the demand-based policy on the facility input of care institutions for older people is not significant. It indicates that the consistency of the internal indicators of the demand-based policy still needs to be improved. The balance between supply-based policy and environmental policy has a negative impact on the facility input of care institutions for older people. It suggests that the balance of the internal indicators of the supply-based and environmental policies can enhance the completeness of the care for older people service system. Rational market supply can inhibit blind investments in care institutions for older people. The balance of demand-based policy has a positive impact on the facility input of care institutions for older people, indicating that the balance of the indicators of demand-based policy gives market supply entities a motivation to invest in institutions' care services.

## Conclusions and Policy Implications

Motivated by the increasing challenges facing by care institutions for older people, the study is aimed to explore the impact of policy instrument mixes on the development of care institutions for older people. It categorizes care policies for older people into three kinds of policy instruments: demand-based, supply-based, and environmental. It measures comprehensiveness, consistency, and balance to characterize the interaction of different policies and the relevance of internal indicators. Thus, a model has been constructed to analyze the impact of the policy instrument mix on the facility input of care institutions for older people.

The main conclusions are as follows: First, the comprehensiveness and consistency of the policy instrument mix showed an increase in 2011–2016, a decline in 2017–2018, and a continued increase in 2019. This is consistent with the trend of changes in the number of care for older people beds in institutions, which shows that there is a certain correlation between the policy instrument mix and the facility input of care institutions for older people. Second, the comprehensiveness and consistency of the policy instrument mix have a positive impact on the facility input of care institutions for older people, and the balance has no significant impact on the facility input of care institutions for older people. It suggests that the facility input of care institutions for older people is significantly affected by policies, and a scientific and reasonable policy combination is conducive to the facility input of care institutions for older people. Third, we focus on optimizing the policy instrument mix to scientifically guide the facility input of care institutions for older people. In the future, the design of care policies for older people should improve the comprehensiveness, consistency, and balance of policy objectives, enhance the coordination of policy instruments, and focus on demand-based policies.

This study analyzes the trends and characteristics of the comprehensiveness, consistency, and balance of elderly service policies promulgated by the Chinese government in 2011–2019, and clarifies the relationship between care for older people service policies and the facility input of care institutions for older people. Our study has a great significance for improving the formulation and improvement of care policies for older people.

The study contributes to the literature on care for older people from the view of policy support. Theoretical research on the policy instrument mix in terms of input of care institutions for older people has been paid attention recently (2, 29). However, studies focusing on the combined effects of different characteristics of a policy instrument mix are scarce (9, 30). This study fills this gap by estimating the influence of the comprehensiveness, consistency, and balance within a policy instrument mix on the develop of institutional care for older people.

Our study shows that the design and implementation of care-related policies for older people affect the resource input of care institutions for older people. Therefore, attaching importance to the scientific setting of policy objectives and the effective combination of policy instruments is conducive to the quality of institutional care. In addition, the actual situation should be fully considered in the design and implementation of policy portfolios. We should pay attention to the coordination and cooperation between supply and demand.

Based on our paper's conclusions, the practical implications are as follows. When the government formulates a policy, the regional environment and care for older people demand preferences need to be considered. It can help improve the resource allocation of care services for older people and improve the utilization rate of care for older people resources. Policy instrument mix can affect the supply of resources to a greater extent. However, from the demand part, such as the bed numbers needed by older people, sometimes are less affected by the policy instrument mix. So, to use the resources more efficiently, the government should fully consider the part of demand part when making policies. In addition, policy consistency and convergence should be paid more attention. For example, many older people in Japan are concerned about healthcare expenditures, the health care system, and health policies. This may be driven by frequent health policy changes and uncertainty owing to regular changes in the administration (9). To avoid vicious competition and help care institutions for older people realize sustainable development, the policy issuers should form industry supervision and information exchange mechanisms. Despite our unique insights, like all research, our study has limitations. Through the process of analyzing the policies, the text mining method is inevitably subjective. And the policies we collected are at the national level, the future studies can also further collect and analyze the policies at the provincial and municipal levels.

## Data Availability Statement

The raw data supporting the conclusions of this article will be made available by the authors, without undue reservation.

## Author Contributions

FZ and CY: conceptualization. FZ and XD: methodology. XiaodY and FZ: validation. FZ: resources. FZ, CY, and XiaotY: writing. XiaodY and QJ: super vision. All authors have read and agreed to the published version of the manuscript.

## Funding

This research was supported by the National Natural Science Foundation (72072142), JSPS KAKENHI Grant (22K01687), the Soft Science Research Program in Shaanxi Province (2022KRM180), the Social Science Fund in Shaanxi Province (2021R034), the Xi'an Soft Science Program (21RKYJ0036), and the Fundamental Research Funds for the Central Universities (SK2022028).

## Conflict of Interest

The authors declare that the research was conducted in the absence of any commercial or financial relationships that could be construed as a potential conflict of interest.

## Publisher's Note

All claims expressed in this article are solely those of the authors and do not necessarily represent those of their affiliated organizations, or those of the publisher, the editors and the reviewers. Any product that may be evaluated in this article, or claim that may be made by its manufacturer, is not guaranteed or endorsed by the publisher.
